# A Taxonomy of DDoS Attack Mitigation Approaches Featured by SDN Technologies in IoT Scenarios

**DOI:** 10.3390/s20113078

**Published:** 2020-05-29

**Authors:** Felipe S. Dantas Silva, Esau Silva, Emidio P. Neto, Marcilio Lemos, Augusto J. Venancio Neto, Flavio Esposito

**Affiliations:** 1 LaTARC Research Lab (IFRN), Federal Institute of Education, Science and Technology of Rio Grande do Norte (IFRN), Natal, RN 59015-000, Brazil; esau.silva@academico.ifrn.edu.br (E.S.); emidio.paiva@ifrn.edu.br (E.P.N.); marciliolemos@ppgsc.ufrn.br (M.L.); 2 Department of Informatics and Applied Mathematics (DIMAp), Federal University of Rio Grande do Norte (UFRN), Natal, RN 59078-970, Brazil; augusto@dimap.ufrn.br; 3 Instituto de Telecomunicações, 3810-193 Aveiro, Portugal; 4 Department of Computer Science, Saint Louis University, Saint Louis, MO 63103, USA; flavio.esposito@slu.edu

**Keywords:** Distributed Denial of Service Attacks (DDoS), Software-Defined Networking (SDN), Internet of Things (IoT), taxonomy, revision, state-of-the-art

## Abstract

The Internet of Things (IoT) has attracted much attention from the Information and Communication Technology (ICT) community in recent years. One of the main reasons for this is the availability of techniques provided by this paradigm, such as environmental monitoring employing user data and everyday objects. The facilities provided by the IoT infrastructure allow the development of a wide range of new business models and applications (e.g., smart homes, smart cities, or e-health). However, there are still concerns over the security measures which need to be addressed to ensure a suitable deployment. Distributed Denial of Service (DDoS) attacks are among the most severe virtual threats at present and occur prominently in this scenario, which can be mainly owed to their ease of execution. In light of this, several research studies have been conducted to find new strategies as well as improve existing techniques and solutions. The use of emerging technologies such as those based on the Software-Defined Networking (SDN) paradigm has proved to be a promising alternative as a means of mitigating DDoS attacks. However, the high granularity that characterizes the IoT scenarios and the wide range of techniques explored during the DDoS attacks make the task of finding and implementing new solutions quite challenging. This problem is exacerbated by the lack of benchmarks that can assist developers when designing new solutions for mitigating DDoS attacks for increasingly complex IoT scenarios. To fill this knowledge gap, in this study we carry out an in-depth investigation of the state-of-the-art and create a taxonomy that describes and characterizes existing solutions and highlights their main limitations. Our taxonomy provides a comprehensive view of the reasons for the deployment of the solutions, and the scenario in which they operate. The results of this study demonstrate the main benefits and drawbacks of each solution set when applied to specific scenarios by examining current trends and future perspectives, for example, the adoption of emerging technologies based on Cloud and Edge (or Fog) Computing.

## 1. Introduction

The Internet of Things (IoT) [1] is expected to cause more changes in the way technology permeates society and the economy. IoT allows everyday objects to be connected to devices that communicate with each other or with other systems via a network. By doing so, IoT enables hyper-connected environments, e.g., in homes, factories, and hospitals, to be leveraged to provide new types of services [2]. However, to ensure the maximum benefits can be derived from this technology, many challenges have to be addressed, particularly in terms of security and privacy [3].

In a typical IoT scenario, there are several smart devices (e.g., sensors, household items, and personal items) that interact with remote applications by collecting and sending data from the environment (e.g., weather information, vital patient data, and traffic information) [4]. Hence, these applications may be vulnerable to the actions of malicious agents that aim to make them unavailable, or even use the IoT devices themselves as a launching pad for attacks on different domains [5]. One of the aggravating factors in this scenario is the lack of embedded security mechanisms, which is often due to the limited processing capacity of the IoT devices [6].

In addition, the large-scale dissemination of new smart devices connected to the Internet is leading to the emergence of increasingly complex IoT infrastructures with heterogeneous features and requirements [7,8]. It is estimated that around 29 billion devices will be connected to the Internet by the year 2022 [9]. These factors make the process of detecting and mitigating virtual threats much more complex and require new defense mechanisms to maintain specialized skills in order to provide an appropriate level of protection.

One of the leading virtual threats today that poses risks to the operation of IoT applications is the Distributed Denial of Service Attack (DDoS) [10]. This type of attack often occurs on the Internet due to its ease of execution (some companies offer DDoS attacks as a service) [11]. In addition, DDoS attacks can threat the availability of a target in seconds. Reports from specialized security companies have revealed a gradual expansion in the scope of DDoS attacks carried out in recent years. The severity of this growth has caused substantial financial losses to corporations and can further affect millions of users worldwide [12]. The most significant DDoS attack ever recorded was directed at the servers of Github in 2018. The attack exploited an area of vulnerability in an application layer protocol that was designed to deliver 129 million requests per second and reach a total volume of traffic of 1.35 Tbps [13], following in the footsteps of the most significant attack recorded in 2016 that involved 1.2 Tbps [14].

### 1.1. Problem Statement

In light of this, there is a need for new technologies that are capable of providing more efficient ways of protecting the network against DDoS attacks. These attacks are usually carried out on a large scale from botnets formed by IoT devices and are mainly aimed at devices that can be exploited [15]. One of the main examples in this regard is the Mirai botnet [16], which in 2016 halted a significant subset of the Internet, impacting several countries. Mirai used an enormous number of geo-distributed IoT devices to orchestrate the attacks. This technique significantly increased the effectiveness of the attack, as it made it difficult to locate the attacker. A recent report [17] shows that in the third quarter of 2019, the United States and the Netherlands had the highest rate of devices owned by botnets that were used for launching DDoS attacks. This problematic situation has required a great effort on the part of the scientific community to investigate new strategies for improving security for IoT infrastructures, intending to mitigate the effects of DDoS attacks.

In this scenario, the advent of new network technologies, such as those brought about by the Software-Defined Networking (SDN) paradigm [18], can be viewed as one of the main advances for reducing the complexity of managing traditional networks [19]. This is made possible through the opportunities provided by the SDN paradigm to design a flexible infrastructure assisted by the capabilities of a programmable control plane. This means that the (network and security) functions that were previously operated individually on each of the network switch can now be carried out and managed in a unified manner through the resources provided by the control plane. Communication between the controller and other network elements is usually carried out through an API that enable the reconfiguration of the system at runtime. Thanks to its (logically) centralized approach, the SDN controller can monitor and manage the entire underlying network infrastructure, and thus provide a holistic view of the network. Concerning security, the devices provided by the SDN architecture can maximize the detection and capacity to contain virtual threats, and thus devise more efficient and programmable mechanisms [20].

Recently, several studies have adopted SDN in the design of techniques for providing security in several sectors, such as access control [21], reliable data communication [22], and malware detection [23,24], among others. Regarding DDoS attacks in particular [25], the solutions that made use of the resources provided by the SDN architecture, proved to be effective in detecting and mitigating attacks, owing to the benefits of programmability introduced through softwarization techniques [26]. These techniques lead to the development of more efficient models that are capable of supporting the constantly evolving DDoS attacks [27].

Thus, there have been several research endeavors aimed at finding new solutions for mitigating DDoS attacks on IoT infrastructures [28]. However, these solutions are tailored on specific situations (e.g., small network infrastructures composed by domestic IoT devices) which prevents these mechanisms from being widely adopted in heterogeneous environments where the requirements are constantly changing. For this reason, it is important to have a holistic understanding of the current DDoS mitigation solutions in the literature. Studies of this nature give insights to both network operators seeking an appropriate mitigation system for their infrastructure and to developers concerned with implementing new mitigation mechanisms. Recent surveys fail to focus on the field of mitigating DDoS attacks harnessing SDN. This knowledge gap hinders our ability to research novel and effective solutions driven by SDN features. Aside from that, the knowledge gap also limits the understanding about the effects of defense mechanisms in the face of certain threat situations resulting from the wide diversity of IoT environments.

The herein above-elicited issues motivate our investigations on chasing to close such a knowledge gap. Hence, the main goal of this paper is to enable security-enhanced IoT environments so that mitigating DDoS attacks through the assistance of SDN features. In light of this main goal, it is imperative to follow several key objectives: (i) in-depth investigation proposing a taxonomy that covers several dimensions related to defense strategies in network infrastructures; (ii) extensive analysis covering the most common application scenarios and include a comparative and qualitative discussion; (iii) correlation of types of both mitigation approaches adopted and mitigation strategies through the inclusion of a few comparison parameters. To accomplish the objectives mentioned above, we have employed a methodology to support the entire research process, which ranges from the review method to the establishment of the taxonomy. The following sections provide a full description of each of the methodological procedures. It is worth mentioning that practical analysis regarding implementation and simulation of attacks is out of the scope of the present paper, but is a key task in our future research directions.

### 1.2. Contributions

The main research contributions of this study are as follows:

(a) A comprehensive review of DDoS attack mitigation strategies featured by Software-Defined Networking technologies for protecting IoT environments.
(b) A classification of mitigation strategies that includes the following dimensions: IoT application scenario, approach to DDos mitigation, mitigation strategy, types of mitigated attacks, SDN architecture, and assessment methodology.
(c) A summary of the current mitigation techniques that employ SDN technologies for DDoS attack mitigation in an IoT scenario.
(d) A comparison of existing mitigation techniques by employing different state-of-the-art performance metrics to show their suitability for each mitigation strategy.
(e) A discussion on open issues and future research directions in DDoS mitigation through SDN in IoT environments.

### 1.3. Paper Organization

The remainder of this paper is structured as follows: in Section 2 we introduce some concepts of related technologies. In Section 3 we examine key research studies that are related to this paper and draws attention to what is contributed to by our research. In Section 4 we define methodologies and research techniques employed in the review, followed by Section 5, where we carry out an in-depth investigation of the state-of-the-art. In Section 6 we propose our taxonomy on DDoS attack mitigation strategies employing SDN techniques in IoT environments. Finally, in Section 7 we conclude our report with some final considerations and give some suggestions for future work in this area.

## 2. Theoretical Background

We begin this section by providing key definitions concerning security issues in the Internet of Things. We then give an overview of Distributed Denial of Service attacks and conclude the section by explaining how Software-Defined Networking can be employed for mitigating DDoS attacks.

### 2.1. IoT Security

Despite existing prospects for revolutionizing the current computing paradigm, there are still several IoT security issues that require investigation. One of these is the lack of security mechanisms in the devices embedded in the IoT ecosystem. In particular, their restrictions in terms of static configurations and computational resources. These limitations make these devices easy targets for various threats that can be found on the Internet [29]. Currently, the most severe security threat in the IoT is that from DDoS attacks [30], which remains a significant challenge in terms of detection and mitigation.

The wide range of IoT application scenarios has led to several different security requirements, and it is a difficult task to meet all of them. Bawany and Shamsi [31] suggests that the security requirements for IoT applications should be assessed in terms of the impact of the DDoS attacks. Table 1 describes the effects of DDoS attacks on particular types of IoT applications and summarizes the security requirements for each application context [31].

The list of stringent requirements that need to honor makes critical-mission verticals (e.g., transportation, healthcare, energy, and others) dependable by nature since failure is not an option. Aside from that, mitigation mechanisms should be proactive and highly effective in chasing the prevention of any type of service disruption events. Although they have significant social implications, applications such as location systems, agricultural automation, and industrial systems have a higher fault tolerance than essential services (e.g., smart grids and healthcare). Furthermore, applications for home automation, water supply, and parking control do not need such strict security standards as the previous two (2) categories [31].

### 2.2. Distributed Denial of Service Attacks

The primary purpose of a Distributed Denial of Service Attack is to deplete the network resources or hardware of a victim (usually an Internet service) and thus make it impossible for legitimate users to access it. In this way, DDoS attacks entail seizing control of various devices and making use of them, to send numerous requests to the victim or to exploit known vulnerabilities [28]. DDoS attacks can be divided into three (3) types: (i) application-layer attacks; (ii) resource exhaustion attacks; and (iii) volumetric attacks [32].

#### 2.2.1. Application Layer Attacks

Attacks on the application layer attempt to take advantage of vulnerabilities that are present in an application or service and can cause instability, thus making it unfeasible for legitimate users to gain access. These attacks are often mistaken for implementation errors, as low rates of malicious traffic are needed to reproduce the behavior of legitimate customers. Thus, these attacks go unnoticed by most of the conventional detection mechanisms [26].

A typical attack on the application layer is the Slowloris [33]. In this situation, incomplete requests are sent to a web server at predefined intervals, to keep several connections open for the longest possible time and, hence, reach the limit for connections that the server can maintain. This means that shortly after the attack begins, the server becomes unable to receive new connections and then becomes unavailable to legitimate users. To ensure it is effective, the Slowloris attack uses the smallest amount of bandwidth possible, which allows it to go unnoticed by detection mechanisms triggered by anomalies [34] in network traffic.

#### 2.2.2. Resource Exhaustion Attacks

Attacks in this category aim to deplete hardware resources such as memory, CPU, and storage, and thus make servers unavailable by exploiting vulnerabilities in protocols that are usually implemented at the network layer. Therefore, exhaustion attacks not only rely on the volume of traffic used but on the combination of specific messages [32].

The principal resource exhaustion attacks involve exploiting the characteristics of the TCP communication protocol. A typical example of this attack is the TCP SYN Flood, which establishes a three-way connection of the TCP protocol to exhaust the space for managing connections (backlog). TCP SYN Flood is performed by sending SYN messages to the victim, using spoofed source addresses [35], and thus always making the target establish a new connection for the malicious customer. Then, the target server waits for confirmation from the client to complete the establishment of the connection, which never occurs. Finally, it causes a depletion of the backlog and, hence makes it impossible to open new connections.

#### 2.2.3. Volumetric Attacks

Volumetric attacks aim to make a system unavailable by saturating the communication links used to access the victim. For this reason, volumetric attacks are much more expressive concerning the amount of traffic generated during their execution when compared to application layer attacks and resource exhaustion or protocol exploitation [36].

The most common volumetric attacks exploit any excessive increase in packet size using the UDP protocol. Well-known examples of volumetric attacks are amplification attacks [37], which send requests to servers on the Internet to alter the source address field with the victim’s address. In essence, this causes the responses to be amplified by the servers and thus exhaust the bandwidth of the target. For this reason, NTP and DNS servers are mainly used as enablers, as they allow high rates of response amplification [38].

### 2.3. Mitigation Strategies for DDoS Attacks by Means of SDN

The benefits introduced by the SDN paradigm, such as the programmability of the control plane and the ubiquitous management of the network, is encouraging the development of new techniques for mitigating virtual threats. The following sections describe the main strategies for mitigating DDoS attacks that employ the SDN paradigm.

#### 2.3.1. Flow Filtering

The flow filtering strategy is implemented natively in OpenFlow-compliant devices, which makes it the most straightforward approach that can be adopted for SDN-based mitigation solutions [39]. This strategy takes into account the fields present in the headers of packages that arrive at OpenFlow devices to block flows classified as malicious. Among the main parameters that can be used during the filtering process are: (i) the source address; (ii) destination address; (iii) port of origin; (iv) destination port; and (v) network layer protocol [40]. Although it is one of the most practical alternatives to implement, it still depends on the gathering of statistics by the controller as well as on the inspection of packets. This approach can result in a considerable increase in delay while detecting the DDoS attack, which could create a bottleneck in the controller communication interface [41].

#### 2.3.2. Honeypots

This strategy involves using systems in isolated and monitored environments that simulate the characteristics of a legitimate target so that information can be collected to update the current detection and mitigation policies. A honeypot is made available on the Internet, which can be hacked by malicious agents who think they are attacking a real target. Although this is a traditional attack mitigation technique, it can be used in combination with SDN, to assist the controller in gathering information about malicious traffic [42].

#### 2.3.3. Rate Limiting

A network may become unavailable as a result of overload in its communication links, which is usually caused by injecting a large amount of malicious traffic, as in the case of volumetric attacks. In this scenario, the SDN controller can define a maximum limit for the volume of traffic that can be processed by the network without the network becoming overloaded. If it reaches the threshold, the network rejects all subsequent packages. Furthermore, security applications usually adopt this strategy in conjunction with Deep Package Inspection procedures [25].

#### 2.3.4. Moving Target Defense

The Moving Target Defense approach entails using techniques to dynamically and continuously reconfigure/update the characteristics of a network or system based on a set of random values to attempt to prevent attackers from making the target system unavailable [43]. One of the main techniques used is the randomization of IP and MAC addresses, which makes it difficult to discover information about hosts and services on the network during the process and prevent possible DDoS attacks. Although the use of MTD techniques has been broadly adopted to mitigate DDoS attacks [43], some gaps related to these mechanisms are considered critical [44], such as the impact in terms of performance and cost caused by the deployment of MTD mechanisms on large-scale networks.

#### 2.3.5. Traceback

Traceback uses the information in packet headers to define an attacker’s real origin. In traditional networks, the process is complicated because the network switches are unable to identify the origin of the packets because the source address field has been falsified. However, the benefits provided by the holistic view of the SDN control plane show the value of finding mitigation solutions based on traceback [45].

#### 2.3.6. Request Prioritization

In this strategy, a priority value is defined for processing the flows that reach the network. The priority works by assigning a default reliability value to the source hosts for each new packet-in that arrives at the SDN controller. The reliability value is based on the traffic history of each host. This value may vary over time as a result of suspicious activity (e.g., when a large number of flows is generated in short time intervals), and this results in a reduced reliability value. In addition, if the source has a low confidentiality value (which is defined by the system administrator), its flows are rejected.

## 3. Related Work

The literature reveals that many studies have been carried out in recent years to foster discussion on general and specific security issues in IoT networks. In the following, we encourage discussion on a set of recent studies in this regard and raise our findings on the analysis.

Kouicem et al. [46] conducted a study on general aspects of IoT security, i.e., confidentiality, privacy, availability, and mitigation of DDoS attacks. In this study, the authors highlighted specific applications in e-health scenarios, smart grids, and smart cities. As a result of their analyses, the solutions reviewed were classified, and took into account factors based on the adoption of emerging technologies such as blockchain and SDN.

In Cherian and Chatterjee [29], the authors reviewed several mechanisms designed to solve problems related to general aspects of information security, and also examined emerging technologies such as SDN, blockchain, and Machine Learning [47].

Farris et al. [48] analyzed several attacks that can compromise the security of IoT devices, such as the spreading of malicious code, DDoS attacks, and attacks on routing devices. In addition, there is a comprehensive discussion of the benefits provided by the Network Function Virtualization (NFV) and SDN technologies for finding solutions for the mitigation of the investigated threats.

The reviews carried out in Kalkan and Zeadally [49] and Kanagavelu and Aung [27] provide an overview of solutions that use SDN to protect IoT infrastructures. The authors held discussions about the analyzed studies and examined the question from the perspective of making a comparison between their advantages and disadvantages.

Lohachab and Karambir [28], Vishwakarma and Jain [26], and Salim et al. [50] created taxonomies that examine existing DDoS attacks, and assessed their impact in different layers of the IoT infrastructure. In addition, the studies reviewed several mechanisms for detecting and mitigating DDoS attacks with a focus on schemes that address technologies such as machine learning, blockchain, and SDN.

The analysis of the related literature reveals that several previous studies have dealt with the question of how to mitigate DDoS attacks in IoT scenarios by employing SDN technologies. However, the authors decided to conduct their studies in a more all-inclusive context and ignored specific features of the scenario under investigation. Table 2 summarizes the contributions made by previous studies and compares them with the proposal outlined here. The comparison is made based on four (4) parameters that are defined in line with the related studies to ensure a fair comparison. The comparative items are described below:

1. Number of solutions reviewed: the higher the number of mitigation solutions included in a study, the more the sample can form the basis of information necessary to assist in understanding the pattern of current mitigation solutions;
2. Classification of mitigation solutions: the classification of solutions provides a means of making it easier to understand how each solution implements its mitigation process and, hence, can characterize the current mitigation of DDoS attacks in IoT scenarios;
3. Comparison of mitigation solutions: a comparison of mitigation solutions is essential for selecting the mechanisms that can be best adapted to the needs of heterogeneous scenarios;
4. Identification of future trends: the identification of promising strategies for the development of future mitigation mechanisms helps researchers who are concerned with finding new mitigation solutions to select the techniques that best suit their specific needs.

The results shown in Table 2 reveal a significant disparity between the related works about this scheme. It is evident that the main objective of previous studies was not to carry out a comprehensive study of DDoS mitigation measures in IoT environments that make use of SDN technologies. Furthermore, none of these previous studies encouraged any extensive discussion of how to provide a classification and create a taxonomy capable of describing and characterizing the solutions under investigation.

## 4. Review Research Method

**Figure 1:**
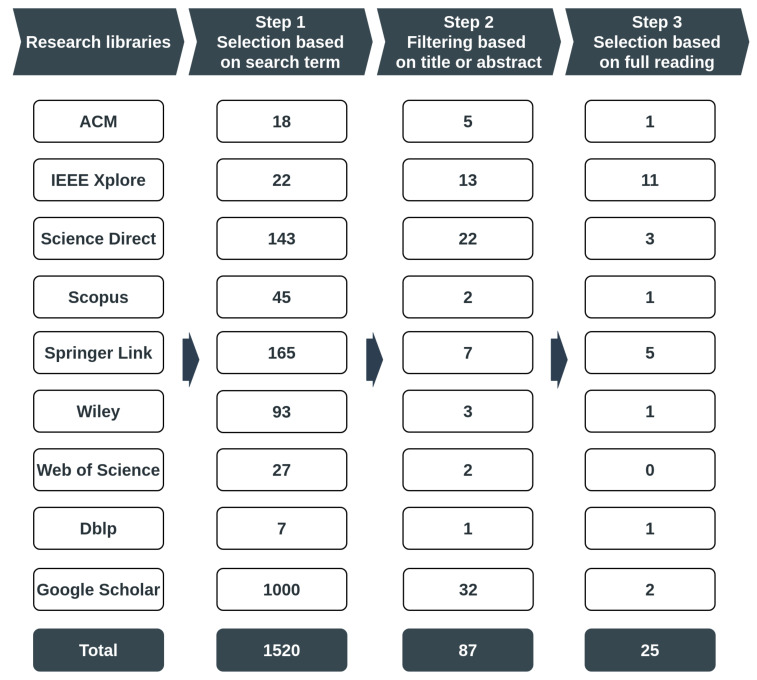
Flowchart for the selection of papers.

The search strategy used in this work followed the method employed by Aleesa et al. [52], as outlined in Figure 1. This includes the following four (4) stages:

1. Selection of the appropriate digital libraries to carry out the search;
2. Definition of a search term based on keywords related to the subject under study;
3. Selection of studies recovered automatically from digital libraries. The decision to include and exclude each work retrieved is based on their titles and abstracts;
4. Selection process in which each study retrieved from the initial selection is fully read and analyzed.

Other factors that may be noted when conducting a literature review, such as the research question, were not regarded as essential for carrying out this work and have, therefore, not been included in the methodology. The searches were conducted between February 2019 and March 2020. Figure 1 outlines the steps taken to follow the search strategy adopted for this study. The results are shown in Figure 1 also include the number of studies selected in each stage. For a fuller understanding, in the following section, we provide details about how the research strategy was implemented.

### 4.1. Selecting Digital Libraries

As shown in Figure 1, the search process has been conducted in the most relevant digital libraries in computer science and engineering, namely:

1. ACM Digital Library (https://dl.acm.org/);
2. IEEE Xplore (https://ieeexplore.ieee.org/);
3. Science Direct (https://www.sciencedirect.com/);
4. Scopus (https://www.scopus.com/);
5. Springer Link (https://link.springer.com/);
6. Web of Science (https://apps.webofknowledge.com/);
7. Wiley (https://onlinelibrary.wiley.com/);

In addition to the studies from the above mentioned digital libraries, there are other relevant studies have been identified using the following automatic search engines:

1. Dblp (https://dblp.uni-trier.de/);
2. Google Scholar (https://scholar.google.com);

The criterion for source selection was the relevance of the open bibliographical information in major areas of computer science, as well as engineering journals and conference proceedings, where high-impact scientific production can be found [39,53].

### 4.2. Search Term Used for the Filter

The search term used in this work has been defined on the basis of a combination of keywords related to the subject under study. The generic form of the search term was based on the acronyms “IOT” AND “SDN” AND “DDOS”, used to retrieve the maximum number of potential key studies. This term was finally translated into the formats accepted by each library.

### 4.3. Filtering Based on Titles and Abstracts

The titles and abstracts of all the documents from digital libraries and search engines were analyzed during the initial selection process based on a set of study selection criteria. This set includes the inclusion and exclusion criteria described below:

- *Inclusion criteria*: (i) studies reporting IoT network security approaches using SDN; (ii) studies addressing DDoS attacks on IoT-SDN networks;
- *Exclusion criteria*: (i) studies that cannot be validated in full text; (ii) previous versions of more complete studies; (iii) studies unrelated to the subject of this research; (iv) studies published in any language other than English.

The first inclusion criterion is more comprehensive than the second criterion. This first criterion allows the search to cover studies that address a broader scope of network security in IoT-SDN scenarios, including but not limited to DDoS. In this case, the concluding remarks and related work sections were further analyzed to determine whether a study should be included or excluded.

### 4.4. Selection Based on Full Reading

The second selection process involved reading and analyzing each pre-selected study. During the reading stage, all the relevant information was extracted from the study, including contextualization, problem definition, proposed solution, and evaluation method. Finally, a qualitative analysis of the extracted data was carried out, and the main findings were used as input to outline the proposed taxonomy.

## 5. Comprehensive Review of Selected Publications

An appropriate selection of the mitigation strategy to be adopted in the design of a scheme for mitigating DDoS attacks is of the utmost importance since it determines the pattern of behavior of the solution. For this reason, the following sections examine the solutions reviewed by this study, categorized according to the mitigation strategies employed, namely: (i) Cosine Similarity; (ii) Flow Filtering; (iii) Rate Limiting; (iv) Moving Target Defense; (v) Traceback; (vi) Request Prioritization; and (vii) Collaboration Between Multiple Mitigation Strategies.

### 5.1. Cosine Similarity

Yin et al. [54] introduce the SD-IoT framework. SD-IoT employs an algorithm based on the cosine similarity technique to carry out DDoS attack mitigation. The algorithm in question compares the rate of packets arriving at the controller at predetermined time intervals with a pre-established ideal limit. This approach enables the classification of devices sending several packets that exceed the amount allowed by the network. This solution proved to be effective in mitigating DDoS attacks based on high traffic rates.

### 5.2. Flow Filtering

Xu et al. [55] discuss the constraints imposed on the use of a single controller to manage IoT infrastructures. Furthermore, the authors argue that this centralized approach jeopardizes the scalability of the network. A clear example of this is the fact that each new packet that arrives at the switches must be sent to the controller for the configuration of flow rules. However, if the number of requests is high, the control link between switch and controller can become overloaded. As a result, the controller is not able to process new flow requests and, consequently, the entire network becomes unavailable. The authors designed the Smart Security Mechanism (SSM) to overcome this problem. This mechanism collects statistics regarding the rate of unmatched flows at each switch over time. The collected statistics allow the values for the correspondence limits to be defined. Switches with values below the limit ar classified as suspect. Following this, traffic from the suspicious switches is sent to a security middleware that is responsible for blocking malicious flows.

Özçelik et al. [56] present the Edge-Centric Software-Defined IoT Defense (ECESID) architecture. ECESID was designed to mitigate DDoS attacks originating from IoT devices infected by malicious agents, such as the Mirai botnet. According to the authors, the closer to the source, the lower the cost incurred for detecting and mitigating attacks. For this reason, the architecture is based on the deployment of small infrastructures close to the protected networks. These infrastructures can analyze all outgoing traffic and check if there are any infected devices. This task is carried out through the Threshold Random Walk employing the Credit-Based Rate Limiting (TRW-CB) algorithm. TRW-CB is based on the number of unsuccessful connection attempts per second. Then, traffic from infected devices is completely blocked.

Salva-Garcia et al. [40] and Molina Zarca et al. [57] introduce a security architecture for large-scale wireless networks based on three (3) planes: (i) Admin Plane; (ii) Security Orchestration Plane; and (iii) Security Enforcement Plane. The mitigation process implements policies defined by the network administrator through the Admin Plane. These policies are then translated into low-level configurations and forwarded to the Security Orchestration Plane. The Security Orchestration Plane is also responsible for monitoring the network, detecting attacks based on a traffic signature database, and selecting the appropriate security policy to mitigate the attack. After selecting the mitigation policy, the Security Enforcement Plane installs it on the SDN switches located at the edge of the network.

Another group of studies proposes architectures with a focus on mitigating DDoS attacks on large-scale networks. These solutions, proposed by Yan et al. [42], Nguyen et al. [58], and Rathore et al. [59], use distributed controllers to address the centralized management limitations of the traditional SDN architecture. These architectures are divided into the following:

- Edge: composed of devices responsible for ensuring access control, secure data communication, and the application of security rules for managing local area networks;
- Fog: composed of SDN controllers responsible for detecting threats, defining the appropriate mitigation policies, and sending them to the devices at the edge layer. In addition, they periodically report the records of events that have occurred to the cloud layer;
- Cloud: contains a holistic view of all managed IoT domains and is, therefore, able to identify attack patterns from different domains. In this way, mitigation policies at the global level can be established.

Yang et al. [60] propose a distributed mitigation architecture that makes use of SDN-based IoT gateways deployed at the edge of the network. The gateways are designed with enhanced capabilities for detecting and mitigating DDoS attacks. SDNIGWs are composed of three (3) modules: (i) Learning Module; (ii) Detection Module, and; (iii) Flow Management Module. The Learning Module collects flow statistics and uses them to train the machine learning-based classification algorithm. The Detection Module performs the classification of malicious flows and, if so, sends the source addresses to be blocked using the filter rules defined by the Flow Management Module.

Rafique et al. [61] address the problem of control-link overhead between switches and controllers, as discussed in Xu et al. [55]. In this context, the authors introduce the SDIoT-Edge Security (SIESec) framework. SIESec makes use of the Edge Computing [62] paradigm to deploy cloudlets close to IoT networks. Each cloudlet implements an instance of SIESec and carries out the detection and mitigation of DDoS attacks by reducing the processing load on the IoT infrastructure. SIESec is composed of six (6) modules: (i) Collector; (ii) Packet Inspector; (iii) Feature Extraction; (iv) Classifier; (v) Status Analyzer; and (vi) Rule Generator. Initially, the Collector module continuously monitors the traffic at each switch and sends traffic statistics to the Packet Inspector and Feature Extraction. These modules pre-process each flow and then send samples from the traffic to the Classifier, which uses the Self Organization Maps (SOM) algorithm to classify each flow as either malicious or benign. The Status Analyzer then forwards the benign flows to their destination, and the malicious flows to be analyzed by the Rule Generator, which is responsible for defining which appropriate filtering rules should be applied.

Krishnan et al. [63] set out a multi-layer architecture called Advanced MultiplAne SecuRity Framework for Software Defined Networks (VARMAN), which is designed for the detection and mitigation of DDoS attacks in SDN-based IoT data centers. The detection of malicious flows is aided by several machine-learning algorithms and takes place during the selection and classification stages. The mitigation process makes use of NFV features that assist in the deployment of several virtual nodes with filtering capabilities. Moreover, VARMAN architecture provides load balancing between SDN controllers.

Bawany and Shamsi [31] introduce the SDN-Based Secure and Agile Framework for Protecting Smart Applications (SEAL). The primary purpose of the SEAL is to provide security for IoT application data centers. SEAL can prioritize the mitigation of DDoS attacks for specific applications based on their security requirements and their assessment of the impact that would be caused by their failure. SEAL is also designed to prevent DDoS attacks on the SDN architecture itself. The framework consists of three (3) key modules: (i) A-Defense; (ii) C-Defense; and (iii) D-Defense. The A-Defense module detects and mitigates DDoS attacks at the application level. It takes account of the individual traffic designed for each IoT application and prioritizes the applications by their security requirements, which might be critical, moderate, or low. An entropy-based detection algorithm assists in the mitigation process to determine which malicious flows must be blocked. C-Defense is responsible for performing the load balancing between controllers. Finally, D-Defense is responsible protect the data plane and uses a model based on traffic statistics for this task.

Ravi and Shalinie [64] employ cloud computing techniques in conjunction with SDN to mitigate DDoS attacks on IoT servers. They have introduced a new mechanism called Learning-Driven Detection Mitigation (LEDEM), which detects DDoS attacks with the aid of a semi-supervised machine learning algorithm. The machine learning algorithm is responsible for identifying all the malicious flows that reach the network and informing the controller of the addresses of the customers that can be classified as malicious. Based on this information, the controller can define the appropriate filtering rules.

Nair et al. [65] devise a mitigation mechanism that draws on information about the relationship between IP and MAC addresses to determine the occurrence of DDOS attacks in a short time. The mechanism checks whether a source MAC address is linked to more than one source IP address, as this can indicate the existence of malicious clients with a forged IP. As well as this, the mechanism uses other parameters such as packet delay time, the number of entries in the flow tables, and the average time needed for the receipt of the packets (per second) by the controller to define a threshold value that determines the existence of a DDoS attack.

Houda et al. [66] employ blockchain in conjunction with SDN to design Co-IoT, a secure, low-cost, flexible, and efficient collaboration scheme against DDoS attacks in IoT environments. Co-IoT relies on smart contracts [67] as a way of sharing information about DDoS attacks between underlying SDN domains in a secure and decentralized way. Initially, each SDN domain has to create and make use of a collaboration agreement, which is carried out through a simplified process. After this, two (2) or more SDN domains establish a trust agreement and set up a network of authorized domains. In this way, whenever one of the domains is the victim of a DDoS attack, it transfers a list of malicious addresses to the domain of origin of the attacks to block these devices.

Galeano-Brajones et al. [68] explore the OpenState, an extension of the Openflow protocol that provides SDN switches with the ability to store information about flows already processed, in addition to storing the rules existing in the flow tables. Thus, part of the intelligence related to containing DDoS attacks on IoT networks is transferred to the switches, reducing the controller load. In addition, the authors use the entropy technique to identify malicious traffic by the controller, which defines the appropriate filtering rules to mitigate the attack.

### 5.3. Rate Limiting

Sharma et al. [69] put forward the ShSec, an architecture for protecting small IoT networks. This mechanism is designed to mitigate volumetric DDoS attacks and perform its detection process based on flow samples collected from the network edge SDN switches. This strategy reduces the amount of traffic sent to the controller and assists in preventing overloads. In addition, ShSec uses buffers on each switch as an additional strategy to mitigate the number of legitimate packets dropped in the wake of a DDoS attack.

### 5.4. Moving Target Defense—MTD

Krishnan et al. [70] introduce a distributed and hierarchical architecture for large-scale networks, consisting of four (4) layers, namely: (i) Cloud; (ii) Edge; (iii) SDN; and (iv) IoT. High-level management is conducted from the cloud layer, which defines security policies and forwards them to the edge layer. In the border layer, there are gateways with SDN support, which forward the traffic to the SDN layer. In turn, the SDN layer processes traffic from external domains. The authors carried out a case study on the use of this architecture against HTTP Flooding DDoS attacks. Mitigation takes place by excluding illegitimate connections and changing the target’s IP address. A message is sent to all the customers with advice about the redirection, along with a CAPTCHA-like computational challenge. Since malicious agents are instructed employing pre-defined commands, they are unable to decode the computational challenge and continue to attack the old address. In this way, only legitimate customers remain connected.

### 5.5. Traceback

Chen et al. [45] provide an architecture that is capable of identifying and mitigating DDoS attacks targeted smart city infrastructures as close as possible to their source. The main purpose of the scheme is to create an algorithm that is capable of collecting flow statistics from base stations through a conventional operation. These statistics are then used to define an acceptable traffic limit. Thus, devices that may be generating traffic above the threshold established for a short period are treated as suspicious and added to an anomaly tree. Then, an additional stage of checking the anomaly tree is performed to determine whether the suspect devices are malicious or not. The devices that are classified as malicious may have their traffic blocked, depending on the mitigation strategy selected by the network administrator.

### 5.6. Request Prioritization

Sarwar et al. [71] present FlowJustifier, a request prioritization algorithm based on a trust return value list, which seeks to mitigate the DDoS attacks that target the SDN-based IoT infrastructure control plane. The list employs confidence values to classify users according to their level of confidentiality, which is established based on the records of each user’s network activities. This means that whenever a user’s flow request reaches the controller, the list of confidence values is checked to define the degree of priority required for processing that user’s request. The trust list values are updated by the controller in real-time whenever a new flow request is processed. Thus, it is also possible to block a sender’s flows if the sender starts sending more requests than usual.

### 5.7. Collaboration between Multiple Mitigation Strategies

In addition to the use of specialized techniques for particular scenarios, the related literature revealed the adoption of solutions based on collaboration between different mitigation strategies. Through this kind of integration, it is possible to offer a series of benefits, including more flexible solutions and the ability to mitigate a significantly greater number of DDoS attacks. Concerning this, the main strategies adopted are Traffic filtering, Machine Learning, and Traffic redirection.

Bull et al. [72] set out a distributed, flexible, and modular SDN architecture capable of mitigating a wide range of DDoS attacks at the edge of the network. The architecture is composed of the following layers: (i) IoT Access; (ii) Distribution Layer; and (iii) Control Layer. The IoT Access layer consists of several IoT devices arranged in different domains. In turn, the Distribution Layer is formed of SDN switches that are responsible for monitoring the traffic coming from the devices in the IoT Access layer. At the top of the architecture is the Control Layer, which has a pool of controllers capable of managing traffic statistics from all protected IoT domains. The Control Layer is also responsible for defining policies to contain malicious flows, and Rate Limiting and Flow Filtering strategies are used together to achieve this goal.

Bhunia and Gurusamy [25] design the SoftThings, a distributed architecture for mitigating a wide range of DDoS attacks on small networks. The SoftThings architecture is segmented into four (4) layers: (i) IoT devices; (ii) SDN switches; (iii) SDN cluster controller; and (iii) SDN master controller. A set of IoT networks represents a cluster. Each cluster is assigned an SDN cluster controller that is responsible for its management and for defining local mitigation policies. In this way, each SDN cluster controller must periodically compile a report on the traffic statistics of the managed networks and send it to the SDN master controller, which is the central entity of SoftThings and has an overview of the entire infrastructure. This means it is in a position to define policies based on Flow filtering that will be applied globally.

Sahay et al. [73] introduce a mechanism for protecting communication systems on ships (i.e., a satellite navigation system) that is capable of detecting and mitigating DDoS attacks. The system works through a high-level language used to specify mitigation policies that can be eventually selected by the network administrator and is applicable to several types of DDoS attacks. Filtering and redirection of malicious traffic are available as some of the policies that can be implemented.

Rafique et al. [74] put forward CFADefense, a mechanism for mitigating Crossfire DDoS attacks. The CFADefense architecture is implemented in three (3) modules: (i) Link Selection; (ii) Attack Detection; and (iii) Malicious Flow Interception. In seeking to mitigate the flows from Crossfire Attack, CFADefense monitors the traffic on each link in the network and selects those with the highest utilization rate so that they can be analyzed by Attack Detection. In turn, Attack Detection calculates various statistics associated with the selected links, such as packet loss, jitter, RTT, and throughput to determine the level of congestion. Once a Crossfire attack has been detected, the Malicious Flow Interception redirects the traffic on the target link and enforces the blocking of malicious flows.

Luo et al. [75] provide a defense method based on SDN, MTD, and honeypots that are capable of protecting IoT environments against DDoS attacks. This method implements an MTD strategy, which keeps changing the IP address of the devices to make it difficult for attackers to discover active hosts. As well as this, a second mechanism manages several SDN-based honeypots and replicates the behavior of legitimate IoT devices to discover the activities of the attackers. By this means, the SDN controller can use the information provided by the honeypots to detect and block traffic coming from attackers in real-time.

## 6. Taxonomy of DDoS Attack Migitation Approaches—Supported Featured by SDN Devices Facilities to Defend IoT

In this section, we introduce our taxonomy intending to describe and characterize DDoS attack mitigation approaches to assist the SDN defense of IoT environments. First, there is a compiled classification that includes the following: technology, granularity, a mitigation strategy, and IoT use case. Each scheme is compared on an individual basis with a set of selected parameters from the current literature. From this point on, we outline how solutions need to be improved and examine some of the main factors that have not been addressed. Following this, we describe the main trends in the current state-of-the-art on DDoS mitigation. Finally, we make some suggestions for future research.

### 6.1. Classification of Reviewed Studies

In an attempt order to compile define an appropriate classification for the solutions reviewed in the previous section, this section establishes a taxonomy based on how each study forms a plan for performing the mitigation of a DDoS attack. The following characteristics are taken into account:

1. Whether the mitigation strategy is implemented collaboratively by distributed network elements or a single controller is responsible for the identification, containment, and remediation stages of DDoS mitigation management. The setting for distributed transactions provides greater reliability as having a centralized controller is a notable point of failure in the network and therefore a target for attackers [76];
2. Whether other technologies, apart from SDN, have been used in the DDoS mitigation strategy. Fog computing, for example, has aroused considerable interest in the network security community, since it provides an opportunity to bring mitigation resources closer to the place where the attack is launched;
3. The type of mitigation strategy recommended;
4. Whether the solution has been planned for a particular IoT scenario, in works where the authors do not clearly define an IoT scenario, it can be assumed that the designed solution is intended to be used in any IoT application scenario.

**Figure 2:**
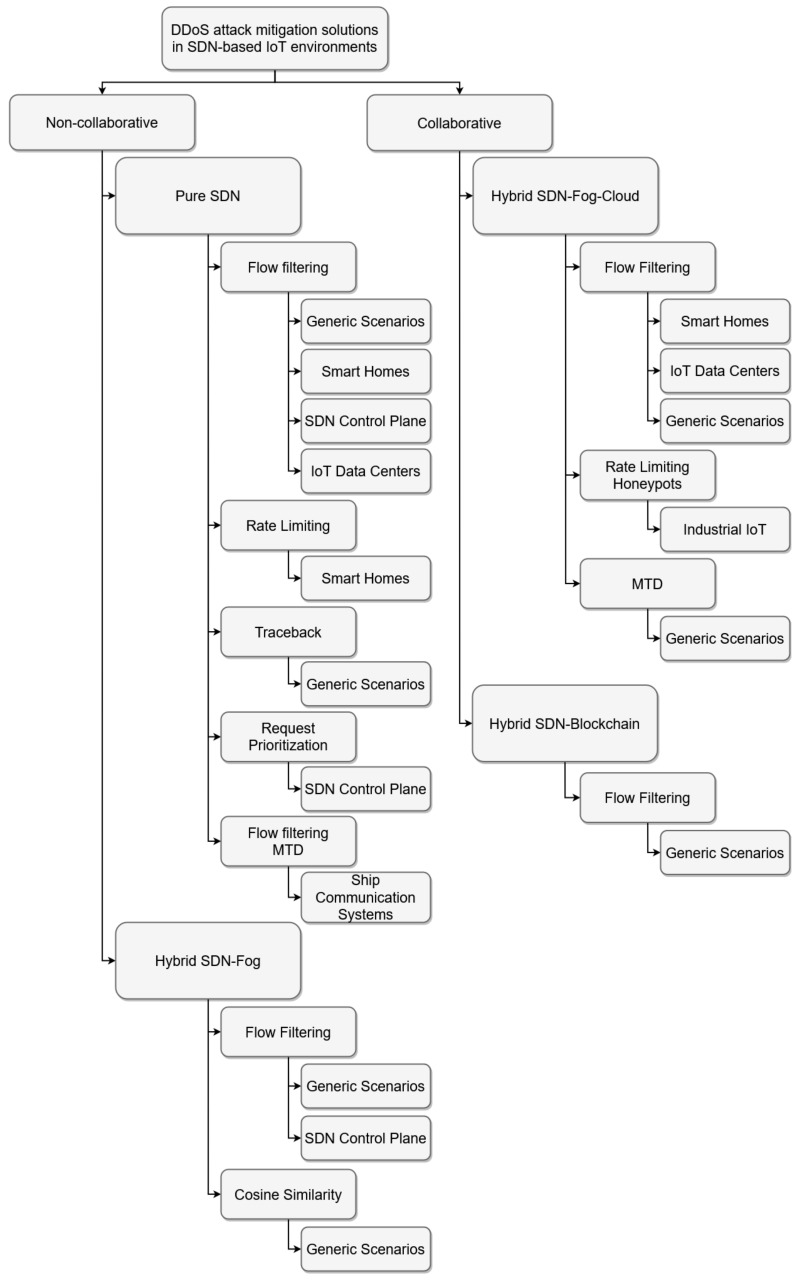
Proposed taxonomy.

Figure 2 outlines these characteristics by creating a taxonomy for the classification of DDoS security solutions in IoT-SDN networks. For a further understanding, the main features of this taxonomy are outlined as follows:

- Non-collaborative solutions: there is no sharing of information (e.g., traffic statistics and malicious source addresses) between network elements (e.g., controllers, IoT gateways with SDN support) that supply the intelligence needed to define and enforce mitigation policies in the SDN-based IoT network;
  - Pure SDN: follows a “pure” (fully-centralized) SDN approach, where a single controller is responsible for responding to DDoS threats;
  - Hybrid SDN-Fog: combines centralized SDN and distributed fog computing in a hybrid design that selects the best features of the two (2) paradigms.
- Collaborative Solutions: the controller must exchange information with external systems to implement a coordinated DDoS mitigation strategy.
  - Hybrid SDN-Fog-Cloud: establishes a multi-layer architecture that encompasses cloud-computing and fog-computing layers. The fog-layers provide an infrastructure to mitigate attacks near the edge of the network that is being defended. In addition, these fog-layers send information about security incidents and network traffic to the cloud layer. The cloud layer uses this information to define the global mitigation policies that the fog-layers must implement.
  - Hybrid SDN-Blockchain: leverages blockchain technology to implement DDoS mitigation in a decentralized and reliable manner. The smart contract mechanism ensures that there is a secure collaboration between the distributed SDN controllers and enables them to block malicious flows as close to their source in the network as possible.

### 6.2. Comparison of Analyzed Solutions

To provide an adequate comparison between the mitigation solutions reviewed in the previous Section 5, we established items considered of high relevance to compose a DDoS attack defense system in IoT ecosystems. We considered these items according to previous related works [77], some revised solutions [56], and technical documents, such as RFC 4732 (Internet Denial-of-Service Considerations [78]) and the US-CERT DDoS Quick Guide [79]. In this sense, Table 3 summarizes the solutions comparison results.

The definition of the comparison parameter featuring Table 3 is highlighted in the enumeration outlines below:

1. Mitigation of internal and external incidents: while it is essential to mitigate DDoS attacks from external domains, mitigation solutions must identify compromised devices on their network, especially to prevent from malicious agents to use these devices for performing DDoS attacks targeting domains on the Internet;
2. Mitigation of multiple DDoS attacks types: solutions for mitigating DDoS attacks in IoT environments must consider both conventional attacks (volumetric) as well more sophisticated attacks that aim to exhaust beyond the bandwidth several other computational resources, such as memory and processing of the target applications;
3. Supporting the SDN control plane to prevent overloads: despite providing several benefits for mitigating DDoS attacks in IoT environments, the SDN control plane can become the target of DDoS attacks directed to exhausting its resources, such as (i) link bandwidth between switch and SDN controller; and (ii) the number of rules stored in the flow tables, overloading the TCAM memory of the OpenFlow switches;
4. Collaborative mitigation: performing DDoS mitigation using only a single controller can cause issues regarding network scalability and fault tolerance. For this reason, it is of immense importance to distribute the mitigation process among several entities that can make mitigation collaboratively to improve performance efficiency and avoid single points of failure in the network.

Observing Table 3, none of the conducted studies are able to match all the elicited items. The solutions that employ cloud and fog computing paradigms, in conjunction with SDN, were the that reached closest. In contrast, the solutions that perform the mitigation only at the edge-side of the network, leveraging the traditional SDN approach, are the ones that most limited and do not meet the requirements defined in the present study.

### 6.3. Overview of the DDoS Attack Mitigation Scenario Harnessing SDN in IoT Environments

The adoption of the SDN paradigm to mitigate DDoS attacks in IoT environments is a relatively new research topic. According to studies available in the literature, researchers published the first SDN-IoT mitigation solution in 2016 [72]. Since then, the community has been developed several mitigation mechanisms in this context. In this way, the first solutions developed attempted to address frequent and well-explored attacks in the literature, such as the mitigation of volumetric attacks using the traditional SDN architecture. However, since 2019 solutions have evolved substantially, and new mechanisms based on the fog computing paradigm have started to be employed on the development of distributed architectures capable of mitigating new types of DDoS attacks based on low traffic rates. In light of this, Table 4 summarizes the DDoS types considered by the analyzed solutions.

According to Table 4, we note that volumetric and exhaust attacks received much more attention from mitigation solutions when compared to attacks on the application layer. In this regard, it is crucial to consider that volumetric and exhaustion DDoS attacks are mainly based on directing large amounts of malicious traffic to the target network. This characteristic facilitates its identification by mechanisms that use more uncomplicated mitigation strategies, such as those that establish limits for traffic arriving on the network (whether malicious or not). In addition, attacks aimed at the application layer are based on sending fragmented requests in different parts, such as DDoS attacks with slow requests and responses, which are easily able to disguise themselves between legitimate traffic due to the use of low traffic rates during its execution. Consequently, this feature allows circumventing mechanisms based on the identification of anomalies in network traffic, making the identification of this type of attack more complex.

Attacks aimed at the application layer are undertaken by sending fragmented requests in different parts, such as DDoS attacks with slow requests and responses; however, these are easily distinguished from legitimate traffic because they rely on low traffic rates during their execution. Hence, this feature allows circumventing mechanisms to be put into effect that is based on their ability to detect anomalies in network traffic, and thus make the identification of this type of attack more complex.

The techniques that rely on the analysis of network flow statistics to detect anomalous actions are efficient in containing volumetric attacks, which are based on high traffic rates [80]. Concerning this, there are solutions based on the following strategies: rate limiting, cosine similarity, traceback, and flow prioritization, as outlined in Table 5. However, when there is a need to mitigate DDoS attacks based on the dispatch of small traffic fees to exploit particular vulnerabilities in the services offered by the IoT infrastructure, techniques based on statistical analysis are ineffective. The main reason for this ineffectiveness is that they are based on anomalous behavior in the network traffic and thus unable to trigger the mitigation process. Only Krishnan et al. [63] and Rafique et al. [61] were able to mitigate DDoS attacks with low rates through traffic filtering and by adopting machine learning algorithms.

#### 6.3.1. IoT Application Scenarios

**Figure 3:**
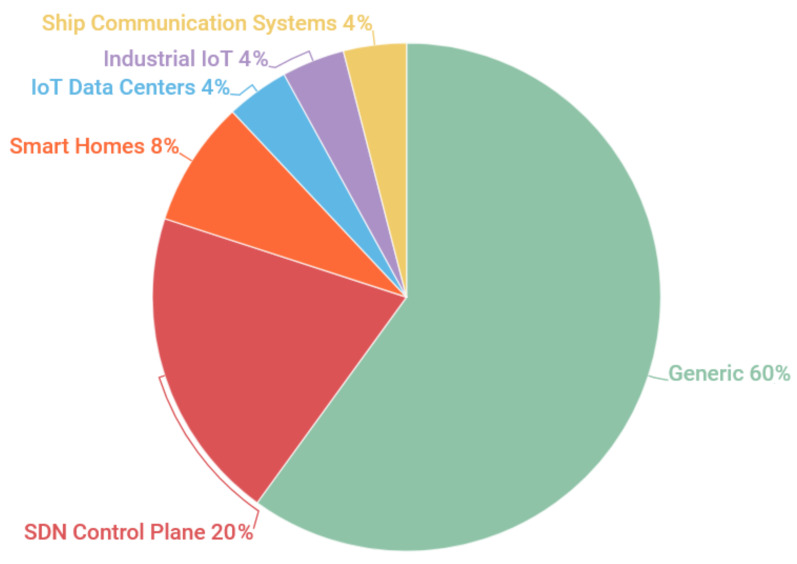
Distribution of mitigation solutions for IoT scenarios.

Figure 3 shows the distribution of mitigation solutions for IoT scenarios. It seems that more than half of the reviewed solutions were not found for a specific IoT scenario. For organizational purposes, we decided to classify mitigation solutions as “generic” when the authors did not clearly define which IoT scenario they were applied to. It was found that mitigation solutions that focused on the SDN control plane and smart homes represented 88% of the solutions reviewed. On the other hand, some IoT scenarios, that had been less explored by the scientific community, had only a single mitigation solution, namely: (i) IoT Data Centers; (ii) Industrial IoT; and (iii) Maritime Communication Infrastructure. As a result, the solutions that focused on these scenarios were grouped as “Other scenarios”, as displayed in Table 6.

#### 6.3.2. Generic

These solutions employ a wide range of methods and architectures to combat different types of resource exhaustion DDoS attacks, as well as mechanisms to detect devices compromised by malicious agents in the local network. In light of this, Özçelik et al. [56] provide an architecture for mitigating DDoS at its origin by adopting fog computing approaches. The adoption of decentralized mitigation approaches that implement secure methods of communication between nodes through emerging technologies such as Blockchain [59] features prominently among the other strategies in this category.

#### 6.3.3. SDN Control Plane

The SDN control plane can itself become the target of DDoS attacks that are aimed at exploiting vulnerabilities in its components and make the underlying IoT applications unavailable. In this scenario, the studies carried out in [55,61,63,71,74] put forward mechanisms that are capable of mitigating DDoS attacks that target both the data plane and the SDN control plane.

Based on the results summarized in Table 3 and Table 4, it is possible to determine the benefits offered by these means of providing security to the SDN control plane that is responsible for accessing IoT applications. In light of this, attention should be drawn to the fact that: (i) the solutions are evaluated against DDoS attacks at low and high traffic rates; and (ii) machine learning approaches are more efficient in detecting malicious flows than conventional mitigation approaches that depend on dynamically-defined traffic limits. These findings corresponded to the solutions by Krishnan et al. [63] and Rafique et al. [61].

In this case, the principal drawback of these solutions that are designed to mitigate DDoS attacks is their fully-centralized approach at the edge of target networks. The main reason for this is that the controller may become unavailable because there is a need to process a huge amount of malicious flows that exceed its computational capacity. Furthermore, solutions in this category were validated in small-scale scenarios. These solutions were only designed to address traffic mitigation from external networks, and all processing of requests and decision-making is centralized in the SDN controller.

#### 6.3.4. Smart Homes

In this category, it should be noted that the main research endeavors are to provide security for both internal and external incidents on the network by using lightweight mechanisms implemented with low-cost hardware (e.g., Raspberry Pi). Among the limitations found in the solutions applied in this scenario are: (i) the mitigation process is implemented centrally at the edge of the network without due concern for the overhead controller; and (ii) the lack of mechanisms capable of mitigating DDoS attacks based on low traffic rates. The use of distributed controllers to perform the mitigation process should be noted as a means of overcoming these limitations in a satisfactory way. In addition, the use of machine learning techniques can be regarded as a feasible alternative, since they are useful in mitigating DDoS attacks based on low traffic rates [63].

#### 6.3.5. Other Scenarios

The mitigation solutions for (i) IoT data centers, (ii) industrial IoT, and (iii) maritime communication infrastructure, have features in common, despite being designed for different scenarios. For this reason, the main benefit offered by solutions in this category is the ability to prioritize the mitigation of DDoS attacks, while taking into account the particular security requirements for each IoT application, as discussed by Bawany and Shamsi [31]. On the other hand, Yan et al. [42] recommend a distributed and scalable architecture that is capable of detecting malicious devices in the protected IoT network itself. The solution found by Sahay et al. [73] rely on traffic filtering based on a static amount of traffic to mitigate DDoS attacks in maritime communication systems. In general, it has been seen that solutions in this category have characteristic limitations, such as (i) their failure to take note of attacks with low traffic rates; and (ii) restrictions on collaboration and maintenance caused by the fact that they were designed for a particular segment.

### 6.4. Open Research Challenges

While the use of the SDN paradigm provides several benefits to mitigate DDoS attacks, in IoT scenarios, several underlying problems have not yet been addressed, namely:

#### 6.4.1. Evaluation of Solutions Based on Realistic Scenarios

According to a recent report [17], DDoS attacks carried out in the third quarter of 2019 reached rates of up to 1Tbps, which shows a sharp rise in the volume of traffic subject to attacks, depending on their type and purpose. Given this, the new schemes must be evaluated in environments capable of replicating as many real conditions as possible, both in terms of traffic and infrastructure. The combination of these factors is crucial to ensuring the scalability of solutions in the face of increasing demand for traffic and the heterogeneity of network infrastructures, currently made up of both physical and virtual elements [19].

#### 6.4.2. Flexibility to Meet Different Security Requirements

IoT ecosystems are made up of a number of applications with different communication and security requirements (e.g., health and disaster recovery applications, traffic control, and smart homes). As a result of the growing demand for mission-critical applications, often sharing resources from general-purpose infrastructures, mitigation mechanisms must be able to identify and prioritize malicious flows intended for this area. In this way, detection and mitigation policies, for example, can be reconfigured at runtime.

#### 6.4.3. Mitigation of DDoS Attacks Based on IoT Protocols

The diversity of the IoT ecosystem offers numerous opportunities for exploring protocols (e.g., MQTT and CoAP), which have been specially designed to meet the demands of new applications. For instance, the CoAP protocol can be used for amplification attacks. Thus, several IoT devices can have their IP addresses spoofed so that attacks can be launched on different domains.

### 6.5. Trends and Opportunities

This section highlights some emerging technologies that provide an opportunity to assist the SDN paradigm in overcoming certain limitations and be able to find more efficient and robust solutions to mitigate DDoS attacks in IoT environments.

#### 6.5.1. Network Function Virtualization

Network Function Virtualization (NFV) [81] is a promising technology when integrated with the SDN paradigm for designing new solutions to mitigate DDoS attacks in IoT environments. NFV introduces a new degree of flexibility and scalability by creating on-demand virtual network appliances such as firewalls, Intrusion Detection Systems (IDS), and Deep Packet Inspection (DPI) Systems. This feature ensures that multiple instances of a specific mitigation mechanism can be implemented precisely at different locations on the network and address the constraints imposed by the occurrence of malicious events [48]. Among the solutions obtained from the use of the integrated SDN and NFV to mitigate DDoS attacks are the schemes employed by Zhou and Guo [82] and Krishnan et al. [63].

#### 6.5.2. Fog Computing

Fog computing is a new architectural concept extending cloud computing, which raise extensive capabilities in affording the discovery of new SDN-based mitigation solutions. Employing fog computing technology allows to access the services offered by the public cloud at a faster data processing speed. Moreover, the deployment of small clouds close to the vicinity of the end-user can improve confidentiality while reduced latency at the same time. The strategies provided in Yan et al. [42] and Özçelik et al. [56] are examples of solutions that harness SDN-based architectures and fog computing to mitigate DDOS attacks in IoT environments.

## 7. Conclusions

This study created a taxonomy to describe and characterize strategies to mitigate DDoS attacks harnessing SDN technologies in IoT environments. As a result, we provide the following contributions: (i) a comprehensive review of the state-of-the-art about DDoS attack mitigation strategies featured by SDN technologies in IoT scenarios; (ii) a new classification guide for the mitigation strategies, which consider several relevant parameters; (iii) a full overview of the existing mitigation techniques for the IoT scenario; (iv) a comparative analysis of the mitigation techniques through pre-defined established criteria supported by related studies, RFCs and other technical documents; and (v) a broad discussion about open issues and research challenges regarding DDoS mitigation featured by SDN technologies in IoT environments.

The proposed taxonomy takes into account four (4) key characteristics associated with the mitigation process, namely: (i) whether the solutions carry out the mitigation process in a centralized or collaborative way; (ii) whether other technologies were used in conjunction with SDN to carry out the mitigation process (hybrid solutions); (iii) the mitigation strategy employed; and (iv) the targeting IoT application scenario.

As a result of a thorough comparison between the analyzed solutions, conducted through a deep investigation in the literature, we note that none of them addressed all the factors that we claim to be regarded as of great significance. However, there were hybrid solutions that exploited the ability of the blockchain, fog, and cloud computing paradigms to provide distributed and highly collaborative solutions, and enabled them to meet the requirements more satisfactorily.

The benefits and drawbacks found in the mitigation solutions that were designed for several different IoT scenarios were also analyzed to assist the scientific community in finding solutions capable of mitigating the most diverse types of DDoS attacks. Thus, solutions that use malicious flow filtering strategies with the aid of machine learning algorithms have proved to be promising in mitigating DDoS attacks when there are both high and low traffic rates.

Finally, a list of new research challenges were identified with potentials to serve as starting points to undertake a new research project. Technologies with a high potential for innovation are also recommended when used in conjunction with SDN.

The findings we obtained from the study carried out in this paper provide the following prospective research directions. We will run practical experiment trials atop a lab-premised testbed, whereby IoT use cases features real system dynamics and varying attack events. Based on the particular resource constraints of the IoT infrastructure, appropriate Key Performance Indicators (KPIs) are needed to provide optimal assessment measuring. The high-accurate analysis and insights from these real trials will potential to drive refinements in our current-proposed taxonomy.

## Figures and Tables

**Table 1:** Classification of Internet of Things (IoT) applications based on your security requirements [31].

IoT Applications	Impact of DDoS Attacks	Security Requirements
Traffic Engineering	High	Services can not be interrupted in any way. Detection with low false-negative rates. Mitigation solutions must be highly proactive.
Electrical network control		
Healthcare		
Location systems	Moderate	Reactive mitigation solutions are the most suitable choice. Detection with low false-negative rates.
Agriculture		
Industrial Management		
Home automation	Low	Reactive approaches are the most appropriate. The allowable interval between detection and the start of the mitigation process is more flexible than in the two (2) previous categories. Low rates of false-positives.
Water supply		
Weather monitoring		
Parking control		

**Table 2:** Analysis of contributions provided by our proposal concerning featured parameters with respect to plain related proposals.

Publication	Year	#1	#2	#3	#4
Kouicem et al. [46]	2018	2			
Kalkan and Zeadally [49]	2018	1			
Lohachab and Karambir [28]	2018	1			
Salman et al. [51]	2018	2			
Kanagavelu and Aung [27]	2019	2			
Cherian and Chatterjee [29]	2019	4	✓		
Vishwakarma and Jain [26]	2019	1	✓		
Salim et al. [50]	2019	4	✓		✓
This work	2020	25	✓	✓	✓

**Table 3:** Comparison of mitigation solutions using Software-Defined Networking (SDN) in IoT environments.

Solution Approach	Mitigation Strategy	Proposal	#1	#2	#3	#4
Pure SDN	Flow filtering	Bull et al. [72]	✓	✓		
		Xu et al. [55]			✓	
		Salva-Garcia et al. [40] & Molina Zarca et al. [57]	✓			
		Rafique et al. [61]		✓		
		Bawany and Shamsi [31]				
		Yang et al. [60]			✓	
		Rafique et al. [74]			✓	
		Nair et al. [65]				
		Galeano-Brajones et al. [68]			✓	
		Ravi and Shalinie [64]			✓	
	Rate limiting	Sharma et al. [69]			✓	
	Traceback	Chen et al. [45]		✓		
	Request prioritization	Sarwar et al. [71]			✓	
	Flow filtering MTD	Sahay et al. [73]		✓		
	Honeypots MTD	Luo et al. [75]		✓		✓
Hybrid SDN-Fog	Cosine similarity	Yin et al. [54]				✓
	Flow filtering	Özçelik et al. [56]	✓	✓		
		Krishnan et al. [63]			✓	
Hybrid SDN-Fog-Cloud	Flow filtering	Bhunia and Gurusamy [25]	✓			✓
		Nguyen et al. [58]		✓	✓	✓
		Rathore et al. [59]		✓	✓	✓
	MTD	Krishnan et al. [70]		✓	✓	✓
	Rate limiting Honeypots	Yan et al. [42]	✓			✓
Hybrid SDN-Blockchain	Traffic filtering	Houda et al. [66]				✓

**Table 4:** Classification of mitigated Distributed Denial of Service (DDoS) attacks type by application scenarios.

		DDoS Attack		
Application Scenario	Proposal	Volumetric	Exhaustion	Application
SDN control plane	Xu et al. [55]		✓	
	Krishnan et al. [63]		✓	✓
	Rafique et al. [61]		✓	✓
	Sarwar et al. [71]		✓	
	Rafique et al. [74]	✓		
Smart Homes	Bhunia and Gurusamy [25]	✓		
	Sharma et al. [69]	✓		
IoT Data Centers	Bawany and Shamsi [31]	✓		
Industrial IoT	Yan et al. [42]		✓	
Ship communication systems	Sahay et al. [73]	✓		
Generic	Bull et al. [72]	✓	✓	
	Özçelik et al. [56]		✓	
	Yin et al. [54]	✓		
	Krishnan et al. [70]		✓	
	Salva-Garcia et al. [40] & Molina Zarca et al. [57]	✓		✓
	Nguyen et al. [58]	✓		
	Rathore et al. [59]	✓	✓	
	Yang et al. [60]	✓		
	Houda et al. [66]	✓		
	Luo et al. [75]	✓		
	Chen et al. [45]	✓		
	Nair et al. [65]	✓		
	Galeano-Brajones et al. [68]	✓		
	Ravi and Shalinie [64]	✓		

**Table 5:** Strategies employed in DDoS mitigation with low and high traffic rate.

DDoS Traffic Rate	Mitigation Strategy
High rate	Honeypots
	Rate limiting
	MTD
	Traceback
	Request prioritization
	Cosine similarity
High and low rate	Traffic filtering with Machine Learning techniques

**Table 6:** Comparison between IoT scenario domains through advantages and disadvantages analysis.

IoT Scenario	Advantages	Disadvantages
Generic	Collaborative Provide secure communication between distributed nodes Mitigation in large and small scenarios Identify malicious devices in the network	Do not consider low traffic rate attacks Validated only in small scenarios
SDN Control Plane	Consider low and high traffic rate attacks	Centralized mitigation on the network edge Non-Collaborative Do not identify malicious devices in the network Validated only in small scenarios
Smart Homes	Lightweight and low cost solutions Identify malicious devices in the network	Centralized mitigation on the network edge Non-Collaborative Validated only in small scenarios Do not consider low traffic rate attacks
Other Scenario	Prioritizes applications by security requirements	Centralized mitigation on the network edge Validated only in small scenarios Do not consider low traffic rate attacks
